# Supplementary material for: Micro-Osteoperforations Induce TNF-α Expression and Accelerate Orthodontic Tooth Movement via TNF-α-Responsive Stromal Cells
Source: Int J Mol Sci. 2022 Mar 9;23(6):2968. doi: 10.3390/ijms23062968 (PMC8955966; doi:10.3390/ijms23062968)
Supplement: Supplementary file 1 [file ijms-23-02968-s001.zip › ijms-1632443-supplementary.pdf]

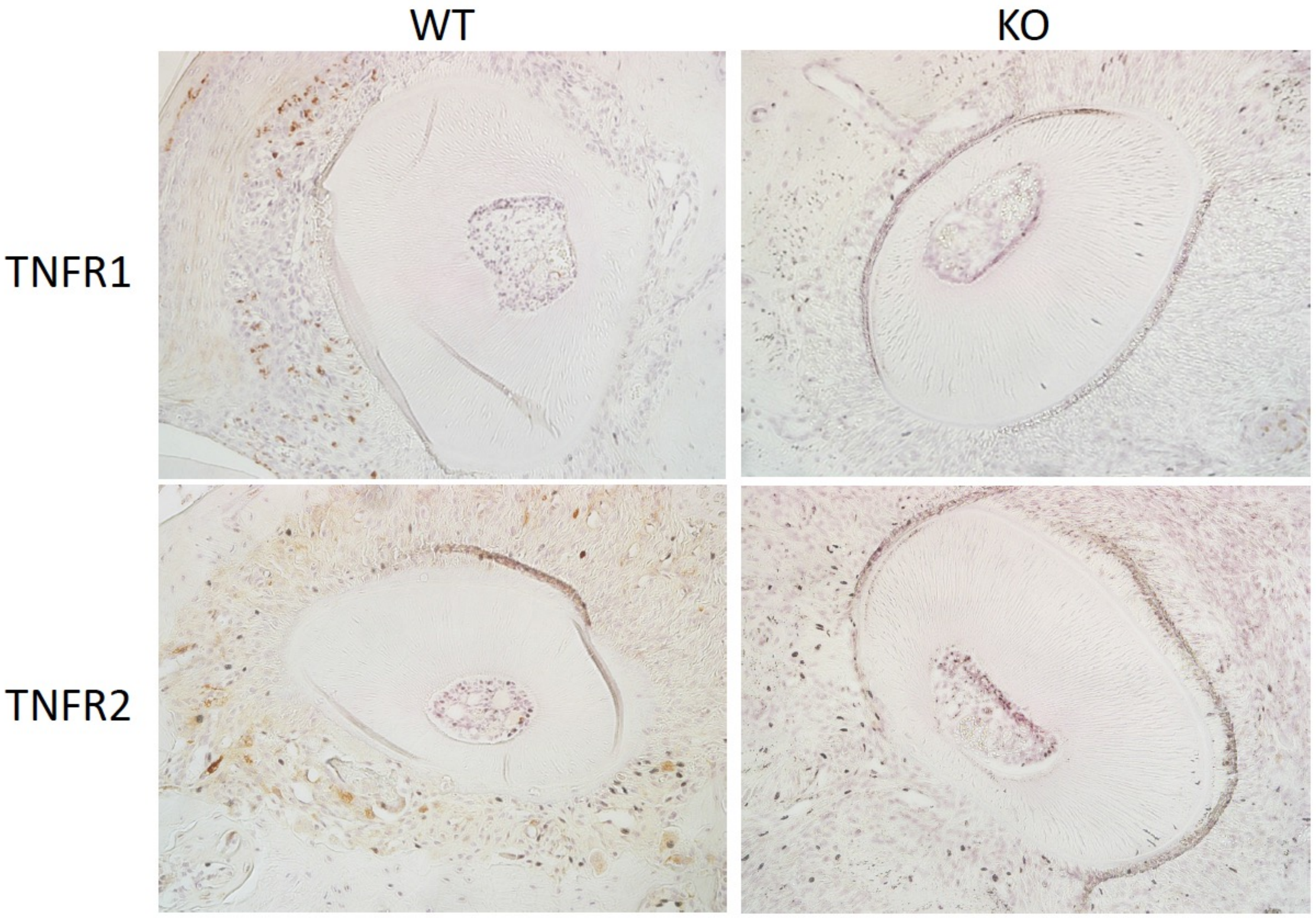

Figure S1. TNFR1 and TNFR2 expressed on PDL cells in WT mice but not TNFRs KO mice. Harvested maxillae from WT and TNFRs KO mice were fixed overnight in 4% paraformaldehyde (diluted in PBS). Samples of maxillae were decalcified in 14% ethylenediaminetetraacetic acid (EDTA) at room temperature for 1 month. The EDTA solution was changed every 2 days. Decalcified maxillae were put in histological cassettes and place them in a bag. Immerse the samples in 1000 mL of 30% ethanol, 70% ethanol, 80% ethanol, and 90%ethanol for 1 h each, 1000 mL of 95% ethanol for 3 hours, 1000 mL of 100% ethanol twice for 7 h and 12 h each, 1000 mL of xylene three times for 0.5 h, 1 h and 1.5 h each for dehydration, and then 1000 mL of liquid paraffin (56 ° C) twice for 7 h and 12 h each in an automatic tissue processor connected to a chemical hood to allow the xylene to evaporate. Horizontal sections of the samples were cut at a thickness of 4 μm. Sections of maxillae were taken at approximately 150 μm from the root branch of the upper-left first molar. For immunohistochemistry, maxillae paraffin sections were deparaffinized, rehydrated, and treated with 0.3%H_2O_2 in PBS for 15 minutes. Sections were then blocked with 5% skim milk for 30 minutes at 37° C and treated with anti-TNFR1 polyclonal antibody (rabbit polyclonal, 21574–1-AP, Proteintech, Rosemont, IL) and anti-TNFR2 polyclonal antibody (rabbit polyclonal, 19272-1-AP, Proteintech, Rosemont, IL) diluted to 1:50 in Can Get Immunostain solution B (Toyobo, Osaka, Japan), overnight at 4° C. Sections were rinsed, then processed with VECTASTAIN Elite ABC Kit PK 6101 (Vector, Burlingame, CA, USA) and treated with 3,3′-diaminobenzidine. Hematoxylin was used for counterstaining. Immunohistochemical analysis showed that there were TNFR1-positive cells and TNFR2-positive cells in the periodontal membrane in WT mice but not TNFRs KO mice.

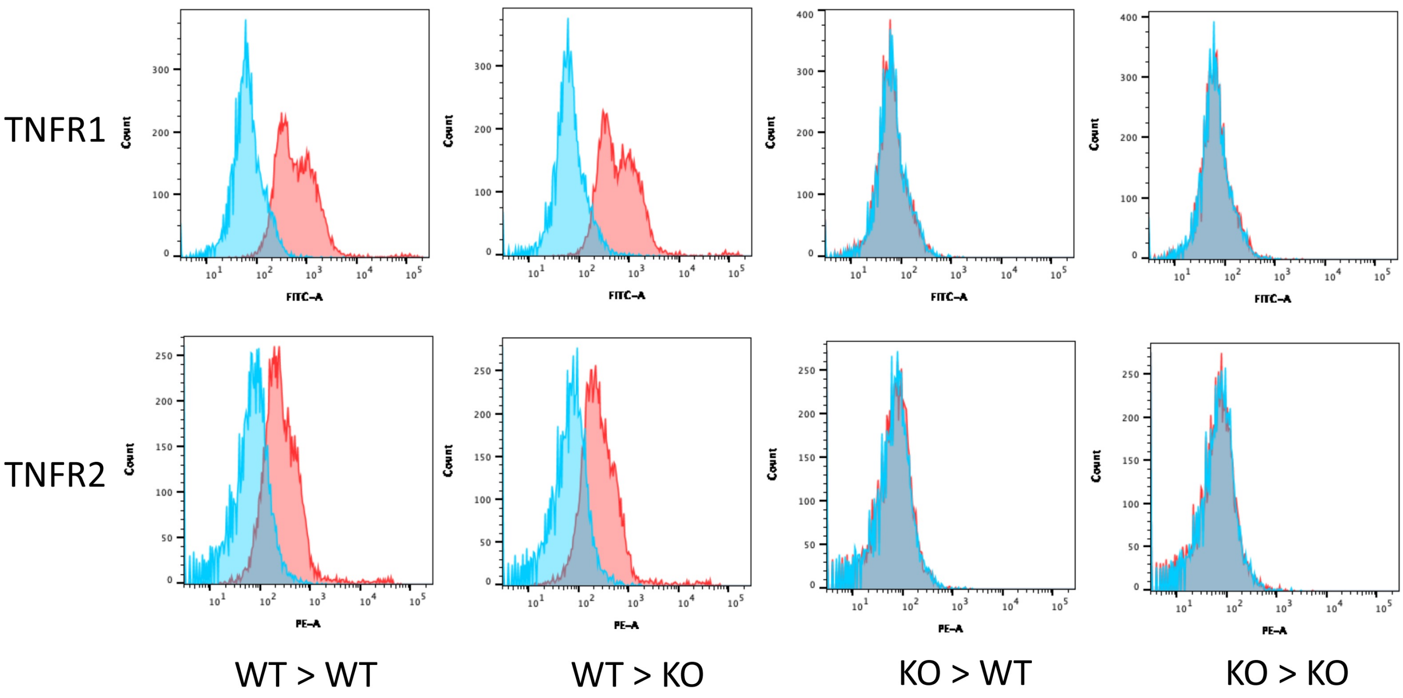

Figure S2. TNFR1 and TNFR2 were selectively expressed on osteoclast precursors in chimeric mice. Bone marrow cells were cultured with M-CSF for 3day. The adherent cells were incubated in NaN3 (0.1%) plus FBS-PBS (FBS, 1%) for 1 hours with FITC-conjugated anti-TNFR1 mAb (Abcam, Cambridge, UK). They were then washed and diluted with NaN3 plus FBS. Furthermore, the cells were also incubated for 1 hour with PE-conjugated anti-TNFR2 mAb (BD Biosciences, San Jose, USA). TNFR1 and TNFR2 expression were analyzed by FACS. Osteoclast precursors of WT>WT and WT>KO expressed TNFR1 and TNFR2, but KO>WT and KO>KO did not express TNFR1 and TNFR2.
